# The Effect of an educational Intervention on Anxiety of Pregnant Women: A Quasi-Experimental Study* ** ***

**DOI:** 10.17533/udea.iee.v40n2e05

**Published:** 2022-09-16

**Authors:** Sajad Pouryousef, Marzieh Kargar Jahromi, Sedigheh Yeganeh, Rouhollah Rouhandeh, Somayeh Paki, Mozhgan Jokar

**Affiliations:** 1 M.Sc of Nursing. School of Nursing, Gerash University of Medical Sciences, Gerash, Iran Email: pourusefsajad@gmail.com School of Nursing Gerash University of Medical Sciences Gerash Iran pourusefsajad@gmail.com; 2 Ph.D Student in Nursing. Student Research Committee, School of Nursing and Midwifery, Shiraz University of Medical Sciences, Shiraz, Iran Email: marzeiah.marziah66@gmail.com School of Nursing and Midwifery Shiraz University of Medical Sciences Shiraz Iran marzeiah.marziah66@gmail.com; 3 M.Sc of nursing. School of Nursing, Gerash University of Medical Sciences, Gerash, Iran Email: sedighe.yegane@yahoo.com School of Nursing Gerash University of Medical Sciences Gerash Iran sedighe.yegane@yahoo.com; 4 M.Sc of Biostatistics. School of Paramedical, Gerash University of Medical Sciences, Gerash, Iran Email: r.rouhandeh@gmail.com School of Paramedical Gerash University of Medical Sciences Gerash Iran r.rouhandeh@gmail.com; 5 M.Sc of nursing. Social Security Organization, Isfahan University of Medical Sciences, Isfahan, Iran Email: s-paki68@yahoo.com Isfahan University of Medical Sciences Isfahan University of Medical Sciences Isfahan Iran s-paki68@yahoo.com; 6 Ph.D Student in Nursing. Student Research Committee, School of Nursing and Midwifery, Shahid Beheshti University of Medical Sciences, Tehran, Iran Email: nurse_jokar@yahoo.com Shahid Beheshti University of Medical Sciences School of Nursing and Midwifery Shahid Beheshti University of Medical Sciences Tehran Iran nurse_jokar@yahoo.com

**Keywords:** anxiety, pregnant women, family nurse practitioners, pregnancy trimester, second., ansiedad, mujeres embarazadas, enfermeras de familia, segundo trimestre del embarazo, ansiedade, gestantes, enfermeiras de saúde da família, segundo trimestre da gravidez

## Abstract

**Objective.:**

The aim of study is the effect of educational intervention on anxiety of pregnant women.

**Methods.:**

This quasi-experimental study is done on the pregnant women referring to family physician’s offices in Gerash City, Iran. 62 women were selected and divided into 2 groups (control and intervention). In intervention group the anxiety reduction training classes were held as a group discussion in 4 weekly 90-minute sessions. Control group received routine care. The anxiety assessment completed by two groups before and after the educational intervention. The measurement instruments included a demographic information questionnaire and the short form of the Pregnancy Related Anxiety Questionnaire (PRAQ-17).

**Results.:**

Comparison of the mean scores of different dimensions of pregnancy anxiety in the pre-intervention and post-intervention stages in the intervention group using paired t-test indicated a statistically significant difference in the dimensions Fear of childbirth, Fear of giving birth to a physically or mentally disabled child, Fear of mood swings and Fear of changes in marital relations (*p* < 0.05) in comparison with control group.

**Conclusion.:**

Holding pregnancy-training classes using group discussion method is a good strategy to reduce anxiety in pregnant women. Therefore, it is recommended that this educational strategy classes be used with mothers from the second trimester of pregnancy in urban family physician centers or those referred to a nearby clinic.

## Resumen

**Objetivo.:**

Evaluar el efecto de una intervención educativa sobre la ansiedad de las mujeres embarazadas.

**Métodos.:**

Estudio cuasi-experimental realizado con la participación de mujeres embarazadas que acuden a las consultas de los médicos de familia en la ciudad de Gerash, Irán. Se seleccionaron 62 mujeres y se dividieron en 2 grupos (control e intervención). En el grupo de intervención, las clases de entrenamiento para la reducción de la ansiedad se impartieron en forma de debate grupal en 4 sesiones semanales de 90 minutos. El grupo de control recibió atención rutinaria. Los dos grupos completaron la evaluación de la ansiedad antes y después de la intervención educativa. Los instrumentos de medición incluían un cuestionario de información sociodemográfica y la forma corta del Cuestionario de Ansiedad Relacionada con el Embarazo (PRAQ-17).

**Resultados.:**

La comparación de las puntuaciones medias de las distintas dimensiones de la ansiedad durante el embarazo en las etapas previa y posterior a la intervención en el grupo de estudio indicó una diferencia estadísticamente significativa en las dimensiones Miedo al parto, Miedo a dar a luz a un niño discapacitado física o mentalmente, Miedo a los cambios de humor, y Miedo a los cambios en las relaciones conyugales (*p* < 0.05), en comparación con el grupo de control.

**Conclusión.:**

La realización de clases de formación durante el embarazo utilizando el método de discusión en grupo es una buena estrategia para reducir la ansiedad en las mujeres embarazadas. Por lo tanto, se recomienda que esta estrategia educativa se emplee con las madres desde el segundo trimestre del embarazo en los centros de medicina de familia o a aquellas que sean derivadas a la consulta externa.

## Resumo

**Objetivo.:**

Avaliar o efeito de uma intervenção educativa sobre a ansiedade em gestantes.

**Métodos.:**

Estudo quase experimental realizado com a participação de gestantes atendidas em consultórios médicos de família na cidade de Gerash, Irã. 62 mulheres foram selecionadas e divididas em 2 grupos (controle e intervenção). No grupo de intervenção, as aulas de treinamento de redução de ansiedade foram ministradas como uma discussão em grupo e em 4 sessões semanais de 90 minutos. O grupo de controle recebeu cuidados de rotina. Ambos os grupos completaram a avaliação da ansiedade antes e após a intervenção educativa. Os instrumentos de medida incluíram um questionário de informações sociodemográficas e a versão curta do Questionário de Ansiedade Relacionada à Gravidez (PRAQ-17).

**Resultados.:**

A comparação das pontuações médias das diferentes dimensões da ansiedade durante a gravidez nas etapas antes e após a intervenção no grupo de estudo indicou diferença estatisticamente significativa nas dimensões; medo do parto, medo de dar à luz um filho com deficiência física ou mental, medo de mudanças de humor e medo de mudanças nas relações conjugais (p < 0,05), em comparação com o grupo de controle.

**Conclusão.:**

A realização de aulas de capacitação durante a gravidez utilizando o método de discussão em grupo é uma boa estratégia para reduzir a ansiedade em gestantes. Portanto, recomenda-se que essa estratégia educativa seja utilizada com mães a partir do segundo trimestre de gestação em centros de medicina de família ou com aquelas que são encaminhadas ao ambulatório.

## Introduction

Despite the advancement of care in the field of physical problems during pregnancy and the change in the method of care provision from health centers to comprehensive centers for urban family physician services, anxiety problems with a prevalence of 54.2% in Iranian pregnant women are still an important issue in the area of health of Iranian pregnant women 1^) (^2 Anxiety is a very unpleasant feeling commonly experienced that is sometimes left undiagnosed or with one or more physical symptoms such as a feeling of emptiness and shortness of breath^3^^) (^4 .Several factors are directly or indirectly involved in the emergence of anxiety during pregnancy. For every woman, certain aspects of pregnancy are considered stressful. 5 These factors include women’s misunderstanding of pregnancy and labor pains, their low age and education, 6 hearing scary stories from others, and the unpleasant experience of previous pregnancies and deliveries. 7 Women’s anxiety during pregnancy can be associated with important consequences, such as improper responses of the mother to the fetus during pregnancy and low birth weight of the child, reduced Apgar scores, postpartum depression, increased risk of preterm birth, and increased risk of neurological and mental illness of the child throughout his/her life 8^) (^10 .

One of the best ways of reducing anxiety in pregnant women and sometimes even their families is attending training classes and group counseling and talking about their concerns with peer groups. 11^) (^13 Pregnancy education in Iran, especially in high-risk populations and subgroups of pregnant mothers, is one of the most important factors effective on the prevention of mortality and reduction of the effects of prenatal complications and depression, anxiety, and subsequent problems. 14 Prenatal training classes provide a great opportunity for mothers to correct their misconceptions and misinformation about pregnancy, delivery, and postpartum issues that cause anxiety among them, and decrease mental tension among women by increasing their understanding of the pregnancy process. 15 In fact, these classes help pregnant mothers to meet other mothers in the same conditions to experience less anxiety and more confidence through familiarization with the resources available in their community. 16 Since quality assurance and improvement of health care for pregnant women is a growing issue in the Iranian health system, and the systematic process of pregnancy care must be continuously and effectively implemented by all members of the family physician team^, (^5 the health service delivery system has been replaced with comprehensive service centers in Fars and Mazandaran Provinces, Iran, since 2012. In the referral system program, the general practitioner and his team (health care provider such as midwife and nurse) have full responsibility for the health of individuals and families covered by them in accordance with a regular care program and, after referring them to specialists, they are responsible for following their condition. 17.

These services include prevention, education, health promotion, and health management services in the population covered. 18 All pregnant women must enter the health care services provision chain through this team to receive health care services and receive the pregnancy care provided by midwives, nurse and other health care provider. 19In addition, health care supervisors should monitor these services and send the related reports and figures to the health centers on a monthly basis. However, the care services for anxiety in pregnant women are not uniformly available in the Iranian Integrated Health Record System (SIB), and the supplementary care provided for women diagnosed with pregnancy anxiety by the health care provider is in the form of referral to centers with psychiatrists, midwives, or nurse practitioners (NP). However, numerous problems such as overcrowding of health centers, the special condition of pregnant women, and the lack of easy access for women to the centers have prevented them from referring to comprehensive service centers and have limited pregnancy care to addressing physical problems and childbirth awareness. In addition, pregnant and newly delivered women in Iran are often fully supported by their families, and thus, have a greater need for training and counseling than care services from the family physician team. .

Therefore, due to the presence of midwives or NPs in the family physician team as family nurses with the important role of caring for and educating family members, 20the researchers decided to use the group training method in the form of family physicians as an important step in reducing women’s anxiety problems, to find a way to provide emergency care for pregnant mothers. Pregnant mothers are certainly one of the high-risk groups and were under more care and follow-up during the New Coronavirus pandemics. In this important direction in our study, we tried to take a small step for this group thus; the aim of study is the effect of educational intervention on anxiety of pregnant women.

## Methods

This was quasi-experimental study. The statistical population consisted of all eligible pregnant women who referred to the selected family physicians’ offices in Gerash City, Iran from November 1 to December 1, 2021.The study was carried out with the objective to investigate the effect of group training on reducing pregnancy anxiety. After obtaining the necessary permissions from the research and ethics committee of Gerash University of Medical Sciences, Gerash, Iran, a list of family physicians’ offices was prepared, which were randomly divided into 2 groups (intervention and control groups). For this work, the list of all family physicians was written on paper and placed in a box and the paper was randomly removed. Paired numbers were in the control group and odd numbers were in the intervention group. Then, by referring to the family physician’s offices and by examining the electronic records, the pregnant women who had the study inclusion criteria were extracted. Adopting the quota sampling method, 70 pregnant women were selected as the participants. Using the Jokar *et al*. study with 95% confidence interval (CI) and 80% power, the sample size was determined as 31 individuals in each of the intervention and control groups, and considering a 15% loss of subjects, this amount was increased to 35 people in each group. ^()^ In the final analysis, 4 subjects from the intervention group due to irregular attendance in the training classes and 4 subjects from the control group due to lack of access to the questionnaire were excluded from the study and 62 subjects (31 in each group) were examined. The study inclusion criteria were: being pregnant women, willingness to participate in the study, age <35 years old, being in the first and second trimesters of pregnancy, no history of hypertension, lack of gestational diabetes, lack of smoking, no signs of risk of miscarriage, lack of placenta Previa, ectopic pregnancy, bleeding, and mental illness, and being Iranian, and fluency in Persian. The exclusion criteria included any problems or illnesses during pregnancy, such as bleeding, multifetal pregnancy, premature rupture of membranes (PROM), diabetes mellitus (DM), and preeclampsia, and failure to attend the training sessions. .

Data Collection Tools. The data collection tools used included a demographic information questionnaire and the Pregnancy Related Anxiety Questionnaire (PRAQ-17). The demographic information questionnaire included 9 items related to age, gestational age, height, ‎weight, gravidity, pregnancy type, education level, occupation of the pregnant woman, and ‎average monthly family income. ‎The PRAQ-17 was designed by Wendenburg and includes 17 items. The exploratory factor analysis (EFA) of data from this questionnaire showed 5 factors, with the factors indicating fear of childbirth (3 items), fear of giving birth to a physically or mentally disabled child (4 items), fear of changes in marital relations (4 items), fear of mood swings and its consequences on the child (3 items), and self-centered fears or fear of changes in the mother’s personal life (3 items). The total score of this questionnaire is the sum of the scores of all items. The score of each item ranges between 1 and 7, so the total PRAQ-17 score can range from 17 to 119. This questionnaire does not have a cut-off point, but to examine correlations, it has been stated that if an individual has 65-70 percent of the assessed criteria, he/she can be identified as evidently anxious. .

Validity and reliability of the questionnaire. The validity and reliability of the questionnaire were measured by Askarizadeh *et al.* ^()^ The reliability of the questionnaire was confirmed based on a Cronbach’s alpha coefficient of 0.78; the Cronbach’s alpha of the 5 factors ‎ranged between 0.69 and 0.76. The CI of the questionnaire 1 month later in 40 pregnant women ranged from 0.65 to 0.72 (*p* < 0.02), indicating the reliability of the PRAQ-17 over time. .

Intervention method. In addition to receiving routine pregnancy care at the family physician’s office in Gerash, the intervention group participants also took part in pregnancy training classes, but mothers in the control group received only routine pregnancy care. Prior to the intervention, Paper consent containing the information of the study research plan was obtained from pregnant women and the mothers were reassured that continuing to participate in the intervention was optional and the results was confidential. Before the intervention, the pregnant mothers in both groups completed demographic information questionnaire and PRAQ-17. The educational classes were presented in 8-12-person classes in 4 weekly 90-minute sessions. We held meetings with full observance of health protocols, observance of social distance, negative Rapid test for mothers. Classes were also held outdoors. Masks, shields and disinfectants were also given to the mothers. The educational content was based on the guidelines of the Iranian Ministry of Health and Medical Education. 23^) (^25The educational content of the first session included an introduction to physiological changes during pregnancy, weight gain, edema, changes in skin color, and regular monitoring of these changes. The educational content of the second session included familiarization with the advantages and disadvantages of natural childbirth and cesarean section, and explanation on the stages of natural childbirth and methods of reducing pain during childbirth. The educational content of the third session included signs and symptoms of postpartum depression and its prevention and treatment strategies. The educational content of the fourth session included some information on physiological and anatomical changes during pregnancy, training on appropriate situations for sexual activity during pregnancy with the presentation of a chart of these conditions and ways to adapt to them, health education, and explanation on some common misconceptions about sexual activity during pregnancy. The last 30 minutes of each session were devoted to answering the participants’ questions. The sessions were held through lectures, question-and-answer, group discussions, and pamphlets. The PRAQ-17 was again distributed among the both group and completed by them 6 weeks after the end of the training sessions. . 

Ethical Issues. The present study was derived from research study with the code of ethics IR.GERUMS.RES.1396.018, which has been approved by Gerash University of Medical Sciences, Gerash, Iran. To resolve the ethical issues, we provided the training booklet to the control group after the study if they wished and wanted. Informed consent was obtained from all individual participants included in the study. . 

Data Analysis. To analyze the collected data, paired t-test, independent t-test, one-way analysis of variance (ANOVA), and the Pearson correlation coefficient were utilized in SPSS software (version 22; IBM Corporation, Armonk, NY, USA). The significance level was *p* < 0.05. .

## Results

A total of 62 subjects (31 women in each group) participated in the study, and the rate of the clearly anxious women in the whole sample before the intervention was 8.1%. There was no significant relationship between any of the demographic characteristics and questionnaire subgroups in the whole sample before the intervention. The mean age of the women participating in the intervention and control groups was 27.23 ± 5.20 and 26.06 ± 5.51 years, respectively. The mean gestational age of the women participating in the experimental and control groups was 19.00 ± 4.35 and 20.25 ± 3.15 weeks, respectively. The mean body mass index (BMI) of the women participating in the intervention and control groups was 25.71 ± 3.71 and 23.61 ± 4.21 kg/m^2^, respectively. Among the participants, 13 (41.9%) and 16 (51.6%) women, respectively, in the intervention group and control group were primiparous. The rate of wanted pregnancy in the control and intervention groups was equal (74.2%). Moreover, 22.6% and 29.0% of the pregnant women in the intervention and control groups had a university degree, respectively. In the intervention and control groups 80.6% and 87.1% of the subjects were housewives, respectively. The comparison of mean age and gestational age between the control and intervention groups at the beginning of the study did not show a significant difference (*p* > 0.05), but the mean BMI of the pregnant women in the intervention group was significantly higher than that of women in the control group (*p* < 0.05) Table 1 Table 2.

**Table 1:** Comparison of mean scores of demographic information between control and intervention

Variable	Control group	Intervention group	***p* -value**
	Mean ± SD	Mean ± SD	
Age	26.06 ± 5.51	27.23 ± 5.20	0.410
Gestational age	20.25 ± 3.15	19.00 ± 4.35	0.200
BMI	23.61 ± 4.21	25.71 ± 3.71	0.042

SD: Standard deviation; BMI: Body mass index

**Table 2:** Frequency distribution of demographic information of control and intervention groups

Variable		Control group			Intervention group
Gravidity	Primiparous	16	51.6	13	41.9
	Multiparous	15	48.4	18	58.1
Pregnancy type	Intended	23	74.2	23	74.2
	Unintended	8	25.8	8	25.8
Education level	Illiterate	0	0	2	6.5
	Elementary	7	22.6	10	32.3
	Secondary school	3	9.7	7	22.6
	High school	12	38.7	5	16.1
	Academic degree	9	29	7	22.6
Occupation	Housewife	27	87.1	25	80.6
	Employee	3	9.7	6	19.4
	Other	1	3.2	0	0
Average monthly family income	< one million	14	45.2	13	41.9
	1-1.9 million	14	45.2	16	51.6
	2-2.9 million	2	6.5	2	6.5
	≥3 million	1	3.2	0	0

The independent t-test was employed to compare the mean scores of different aspects of pre-intervention pregnancy anxiety between the control and intervention groups. The results suggested that there was no statistically significant difference between the groups in any of the dimensions (fear of childbirth, fear of giving birth to a physically or mentally disabled child, fear of mood swings, fear of changes in marital relations, self-centered fears, and total pregnancy anxiety) (*p* > 0.05) Table 3. .

**Table 3:** Comparison of mean scores of pregnancy anxiety dimensions before and after the intervention in the control and intervention groups

Dimensions of pregnancy anxiety	Group	Pre-intervention Mean ± SD	Post-intervention Mean ± SD	**Independent t-test *p*-value**
Fear of childbirth	Control	9.13 ± 5.85	7.83 ± 4.77	0.214
	Intervention	9.09 ± 5.31	6.00 ± 2.94	0.004
	*p*-value*	0.980	0.066	
Fear of giving birth to a physically or mentally disabled child	Control	8.90 ± 5.83	7.61 ± 4.73	0.327
	Intervention	8.58 ± 5.48	5.64 ± 1.72	0.003
	*p*-value*	0.823	0.033	
Fear of mood swings	Control	8.80 ± 4.32	7.70 ± 4.76	0.219
	Intervention	8.74 ± 4.06	6.77 ± 3.74	0.005
	*p*-value*	0.957	0.331	
Fear of changes in marital relations	Control	9.29 ± 3.90	8.54 ± 3.92	0.383
	Intervention	9.85 ± 3.45	7.93 ± 3.59	0.023
	*p*-value*	0.757	0.524	
Self-centered fears	Control	8.78 ± 4.33	6.90 ± 3.27	0.259
	Intervention	8.77 ± 3.95	8.32 ± 4.26	0.596
	*p*-value*	0.394	0.167	
Total pregnancy anxiety	Control	43.87 ± 18.39	38.77 ± 16.52	0.164
	Intervention	44.77 ± 15.81	34.77 ± 13.90	0.001
	*p*-value*	0.836	0.248	

* Independent t-test

The comparison of the mean scores of different dimensions of post-intervention pregnancy anxiety between the control and intervention groups indicated a decrease in the level of anxiety of pregnant mothers in both groups; however, the level of anxiety in the intervention group showed a greater decrease. The independent t-test showed a statistically significant difference in the mean score of fear of giving birth to a physically or mentally disabled child ‎between the two groups in the post-intervention stage (*p* < 0.05), but there were no significant differences in the other dimensions (fear of childbirth, fear of mood swings, fear of changes in marital relations, self-centered fears, and total pregnancy anxiety) (*p* > 0.05) Table 3. .

Comparison of the mean scores of different dimensions of pregnancy anxiety in the control group before and after the intervention using paired t-test did not show a statistically significant difference (*p* > 0.05) Table 3. Comparison of the mean scores of different dimensions of pregnancy anxiety in the intervention group before and after the intervention using paired t-test showed a statistically significant difference in all dimensions except self-centered fears (*p* < 0.05) Table 3. No significant relationship was observed between the different dimensions of pregnancy anxiety and the demographic information (*p* > 0.05). .

## Discussion

The mean scores of different dimensions of pregnancy anxiety in the intervention group before and after the intervention revealed that the group discussion training method caused a statistically significant difference in all dimensions except self-centered fears. Girija *et al.* also considered group education as one of the main methods to reduce pregnancy anxiety in prim parous women and stated that prenatal group training can significantly reduce women’s anxiety. 26 This results can be due to the use of the experiences of the peer group under the supervision of the instructor and reduction of anxiety during training. 27 Fear of change in marital relations is one of the issues associated with anxiety in pregnant women on which group education had a positive effect (*p* < 0.02). Kazemi *et al* . 28 And Salehi and Shah Hosseini 29 declared that most pregnant Iranian women participating in the study had marital problems related to fear of harm to the fetus and physical problems. Akbarinejad *et al.* stated that, despite the cultural and religious limitations of women in expressing their marital problems, group education provided an opportunity for women in Gerash to reduce their fear of change in marital relations by reducing their pregnancy anxiety. 30 .

In urban family physician programs, pregnancy care is provided by a physician and health care provider such as nurses, while training classes are held at comprehensive health centers. 17According to the most recent statistics announced by the Gerash Health Department, pregnant mothers had the lowest participation rate in educational classes, so that only 8% participated in classes on natural childbirth benefits and 9% in childbirth danger education training class in 2019. Lu *et al.* stated that there is a gap between the current care provided and high-quality care, and that continuous follow-up and clarification can be effective in reducing this gap. In this regard, it can be claimed that the presence of health care providers (including NPs and midwives) alongside family physicians, as one of the main pillars of the referral system, plays a significant role in reducing pregnancy anxiety through their encouragement of pregnant mothers to participate regularly in pregnancy training classes and follow-up on their presence in these classes. Nurses Practitioner and midwife in the hospital can also use this training program to reduce the anxiety of women referring to labor and gynecology wards and even postpartum care. .

## Limitation of the study

This was a quasi-experimental study performed on pregnant women under 35 years of age who were in the first and second trimesters of pregnancy, so it is recommended that subsequent clinical trials be performed on high-risk women (over 35 years of age or lower 18 years of old) with period follow up. .

## Conclusion

Holding pregnancy training classes using group discussion method is a suitable strategy to reduce anxiety in pregnant women covered by an urban family doctor. Therefore, it is recommended that educational classes be continuously held as group discussion in the second trimester of pregnancy in urban family physician centers or mothers be referred to a nearby clinic. The following up and monitoring of these classes by health center education liaisons is also recommended. Therefore, group education under the supervision of a family physician can be used as an acceptable way to reduce anxiety and fear among pregnant women.

## References

[1] Alipour Z, Kheirabadi GR, Eslami AA, Kazemi AJJoE, Promotion H (2018). Psychological profiles of risk for antenatal depression and anxiety in Iranian sociocultural context. J. Educ. Health Promot..

[2] Arfaie K, Nahidi F, Simbar M, Bakhtiari MJEp (2017). The role of fear of childbirth in pregnancy related anxiety in Iranian women: a qualitative research. Electron. Physician..

[3] Imanparast R, Bermas H, Danesh S, Ajoudani ZJSJ (2014). The effect of cognitive behavior therapy on anxiety reduction of first normal vaginal delivery. J. Shahid Sadoughi Univ. Med. Sci.

[4] Bolea-Alamañac B, Davies SJ, Evans J, Joinson C, Pearson R, Skapinakis P (2019). Does maternal somatic anxiety in pregnancy predispose children to hyperactivity?. Eur. Child. Adolesc. Psychiatry.

[5] Haring M, Smith J, Bodnar D, Misri Sh LR, Ryan D (2013). Coping with anxiety during pregnancy and following the birth: A cognitive behaviour therapy based self-management-guide for women and health care providers. The BC Reproductive Mental Health Program.

[6] Silva MMdJ, Nogueira DA, Clapis MJ, Leite E (2017). Anxiety in pregnancy: prevalence and associated factors.. Rev. Esc. Enferm. USP..

[7] Giardinelli L, Innocenti A, Benni L, Stefanini M, Lino G, Lunardi C (2012). Depression and anxiety in perinatal period: prevalence and risk factors in an Italian sample. Arch. Womens Ment. Health.

[8] Chen J, Li Q, Rialdi A, Mystal E, Ly J, Finik J (2014). Influences of maternal stress during pregnancy on the epi/genome: comparison of placenta and umbilical cord blood. J. Depress. Anxiety.

[9] Karimi L, Makvandi S, Mahdavian M, Khalili R. (2021). Relationship between social support and anxiety caused by COVID-19 in pregnant women. Iran. J. Obstet. Gynecol. Infertil.

[10] Andaroon N, Kordi M, Kimiaee SA, Esmaily H. (2018). Effect of Individual Counseling Program by a Midwife on Anxiety during Pregnancy in Nulliparous Women. Iran. J. Obstet. Gynecol. Infertil.

[11] P .Parsa, Saeedzadeh N, Masoumi SZ, Roshanaei G (2016). The Effectiveness of Counseling in Reducing Anxiety Among Nulliparous Pregnant Women. J. Family Reprod. Health..

[12] Alaem F, Jalali A, Almasi A, Abdi A, Khalili M (2019). Investigating the effect of group counseling on family stress and anxiety of primiparous mothers during delivery. Bio liPsychoSocial Med..

[13] Akbarian Z, Kohan S, Nasiri H, Ehsanpour S (2018). The Effects of Mental Health Training Program on Stress, Anxiety, and Depression during Pregnancy.. Iran. J. Nurs. Midwifery Res.

[14] Yousefzadeh S, Dm Esmaeili, Ym Asadi, Mt Shakeri (2016). Effects of Training about the Benefits of Natural Childbirth during Pregnancy on the Attitude and Intentions to Select the Mode of Delivery in Nulliparous Women. J. Midwifery Reprod. Health.

[15] F .Rahimi, Ahmadi M, Rosta F, Majd HA, Valiani MJSP, Prevention I (2014). Effect of relaxation training on pregnancy anxiety in high risk women. J. Saf. Promot. Inj. Prev..

[16] Bazrafshan MR, Ghorbani ZJJoh (2010). The effect of slow stroke back massages on anxiety among primigravid women. J. Hayat.

[17] Damari B, Vosough Moghaddam A, Rostami Gooran N, Kabir MJ (2016). Evaluation of the Urban Family Physician and Referral System Program in Fars and Mazandran Provinces: History, Achievements, Challenges and Solutions. J. Sch. Public Health Inst. Public Health Res.

[18] Ebrahimipour H, Hosseini SE, Mahmoudian P, Vafaee Najar A, Zomorodi Niat H, Emamian HJB (2015). Evaluating the performance of family physician in rural health centers, Bardaskan, Iran.. J. Public Health.

[19] Behzad D, Abbas Vosough M, Narges Rostami G, Mohammad Javad K (2016). Evaluation of the Urban Family Physician and Referral System Program in Fars and Mazandran Provinces: History, Achievements, Challenges and Solutions. J. Sch. Public Health Inst. Public Health Res.

[20] Alizadeh M, Yamani N, Taleghani F, Changiz T (2011). Determining the Professional Tasks of Family Nurses through the Viewpoints of Nurses, Families, Physicians, and Managers. Iran. J. Med. Educ.

[21] Jokar M, Oshvandi K, Seifi Z, Paki SJJoDN (2016). The Effect of Teach-back Communication Strategy on Improving Attitude Components of Type II Diabetic Patients: A Clinical Trial Study. Diabetes Nurs..

[22] G .Askarizadeh, Karamoozian M, Darekordi AJIjopm (2017). Validation of Iranian version of pregnancy related anxiety questionnaire. Int. J. Prev. Med.

[23] Kiasalar M, Madadi Taeme Z (2016). Pregnancy self-care - week by week.

[24] Emami Afshar N, Sh Tork Zahrani, Jalilvand P (2017). Pregnancy training and preparation for childbirth. Pajhwok Arman Publications.

[25] Integrated care for the health of mothers (2016). Author of the Ministry of Health, Treatment and Medical Education, Office of Population Health, Family and Schools, Maternal Health Management.

[26] Madhavanprabhakaran K, D’Souza S, KarkadaSubrahmanya N (2017). Effectiveness of childbirth education on nulliparous women’s knowledge of childbirth preparation, pregnancy anxiety and pregnancy outcomes. Nurs. Midwifery Stud.

[27] Karimi A, Moradi O, Shahoei R (2016). The effect of Teasdales cognitive therapy on anxiety reduction during pregnancy.. J. Mod. Psychol. Res.

[28] Kazemi F, Nahidi F, Kariman N (2017). Exploring factors behind pregnant women's quality of life in Iran: a qualitative study. Electron. Physician..

[29] Salehi F, Shahhosseini Z (2017). Association between Women’s Marital Satisfaction and Anxiety during Pregnancy. Iranian J. Psychiatry Behav. Sci.

[30] Akbarinejad Z, Alidoosti K, Ghorashi Z, Asadollahi Z (2020). The Effect of Psychoeducational Group Counseling on Postnatall Sexual Intimacy of Lactating Women Referring to Urban Health Centers in Rafsanjan City: An Educational Trial. J. Rafsanjan Unive. Med. Sci.

